# l-carnitine alleviates synovitis in knee osteoarthritis by regulating lipid accumulation and mitochondrial function through the AMPK-ACC-CPT1 signaling pathway

**DOI:** 10.1186/s13018-023-03872-9

**Published:** 2023-05-26

**Authors:** Taiyang Liao, Wei Mei, Li Zhang, Liang Ding, Nan Yang, Peimin Wang, Li Zhang

**Affiliations:** 1 Department of Orthopedics, Affiliated Hospital of Nanjing University of Chinese Medicine, Nanjing, 210029 China; 2 Jiangsu Province Hospital of Chinese Medicine, Nanjing, 210029 China; 3 Key Laboratory for Metabolic Diseases in Chinese Medicine, First College of Clinical Medicine, Nanjing University of Chinese Medicine, Nanjing, 210023 China

**Keywords:** Knee osteoarthritis (KOA), l-carnitine, Synovitis, AMPK-ACC-CPT1 pathway, Mitochondrial function, Lipid accumulation

## Abstract

**Background:**

Knee osteoarthritis (KOA) is a disability-associated condition that is rapidly growing with the increase in obesity rates worldwide. There is a pressing need for precise management and timely intervention in the development of KOA. l-carnitine has been frequently recommended as a supplement to increase physical activity in obese individuals due to its role in fatty acid metabolism, immune disorders, and in maintaining the mitochondrial acetyl-CoA/CoA ratio. In this study, we aimed to investigate the anti-inflammatory effects of l-carnitine on KOA and delineate a potential molecular mechanism.

**Methods:**

Lipopolysaccharide-stimulated primary rat fibroblast-like synoviocytes (FLS) were treated with an AMP-activated protein kinase (AMPK) inhibitor or siRNA and carnitine palmitoyltransferase 1 (CPT1) siRNA to examine the synovial protective effects of l-carnitine. An anterior cruciate ligament transection model of rats was treated with an AMPK agonist (metformin) and CPT1 inhibitor (etomoxir) to define the therapeutic effects of l-carnitine.

**Results:**

l-carnitine displayed a protective effect against synovitis of KOA in vitro and in vivo experiments. Specifically, l-carnitine treatment can reduce synovitis by inhibiting AMPK-ACC-CPT1 pathway activation and showed an increase in fatty acid β-oxidation, a lower lipid accumulation, and a noticeable improvement in mitochondrial function.

**Conclusions:**

Our data suggested that l-carnitine can mitigate synovitis in FLS and synovial tissue, and the underlying mechanism may be related to improving mitochondrial function and reducing lipid accumulation via the AMPK-ACC-CPT1 signaling pathway. Therefore, l-carnitine may be a potential treatment strategy for KOA.

## Introduction

Knee osteoarthritis (KOA) is the most common form of arthritis and is characterized by the loss of structure in the articular cartilage, remodeling of the subchondral bone, generation of synovial inflammation, and osteophyte formation [1]. The disease is triggered by a series of factors, with associated causative factors, e.g., obesity, age, trauma, sex, and ethnicity [2, 3]. OA affects 7% of the general population, and it is assessed that 1/3 of people over the age of sixty-five continue to be plagued by the complaint [4, 5]. This matches nearly 500 million individuals, a figure that increased by 48% from 1990 to 2019 in the world [4, 5]. Although osteoarthritis is the leading cause of disability and the 15th highest cause of years lived with disability worldwide, no cure or disease-modifying treatments are available [2, 6]. The nonoperative treatment of KOA mainly includes anti-inflammatory drugs and biologics, such as non-steroidal anti-inflammatory drugs (NSAIDs), hyaluronic acid, glucocorticoids, diacrine, and chondroitin sulfate [7]. However, many people cannot accept the undesired side effects of these drugs such as gastric hemorrhage, perforation of gastric ulcers, and cardiovascular disease [8]. More importantly, exercise-based therapy to reduce body weight for the treatment of KOA is consistently recommended in treatment guidelines [9]. Therefore, more recent research has shifted the focus to screening a drug that targets the lipotoxicity of individuals with fewer side effects to prevent and treat KOA [10, 11].

In the context of obesity, the accumulation of free fatty acids in joints leads to organ lipotoxicity, and rising lipid levels contribute to the occurrence of KOA, with consequent tissue inflammation [12]. Significantly, a major pathway of lipid depletion is mitochondrial β-oxidation, and numerous studies have shown that improving mitochondrial function plays a crucial role in reducing lipid accumulation [13]. It is widely known that mitochondrial dysfunction (high ROS production, apoptotic cells, decreased ΔΨm, and low cellular ATP levels) boosts the pathogenesis of KOA because it involves lipid homeostasis and cytokine release and promotes cell apoptosis [14, 15]. On the other hand, AMPK signaling, a regulator of lipid metabolism and energy homeostasis, has been proven to limit the development and progression of osteoarthritis [16]. Specifically, AMPK can inactivate ACC by phosphorylation, resulting in reduced activity of malonate monoacyl coenzyme A (CoA) and reducing allosteric inhibition of CPT1, thereby enhancing the activity of CPT1, which transports long-chain fatty acids from carnitine to mitochondria for β-oxidation [17]. As the rate-limiting step for β-oxidation, the awakening of CPT1 can help maintain the balance of energy metabolism and lipid metabolism [18]. Moreover, a previous study showed that overexpression of CPT1A can reverse the inflammation and fibrosis status of the kidneys by restoring mitochondrial homeostasis [19]. Thus, the activation of the AMPK-ACC-CPT1 pathway can promote β-oxidation of lipids, but its contribution in KOA still needs to be investigated in depth. l-carnitine, a quaternary ammonium compound, is essential for bioenergetic processes, where it acts as a fatty acid transporter of acyl radicals from the cytoplasm to the intramitochondrial sites for β-oxidation to complete the process of energy metabolism and lipid metabolism [20]. As mentioned above, l-carnitine is needed for CPT1 to work, and the levels of l-carnitine are also related to CPT1 transcription and ketogenesis. In addition, cumulative evidence suggests that KOA as a metabolic disorder also requires energy metabolic pathways to meet the demands of tissue repair; thus, l-carnitine shines in the area of energy supplementation [21]. A recent study demonstrated that l-carnitine supplementation can reduce synovial inflammation and cartilage destruction and alleviate pain at the central and peripheral levels [22]. Our previous work also addressed the role of fibroblast-like synoviocytes (FLS) in AMPK signaling during KOA, accordingly suggesting that these cells promote both the onset and the progression of synovitis by regulating AMPK Signaling [23]. When LPS-induced inflammatory responses in FLS occur, TRPA1-mediated Ca^2+^ influx is enhanced and IL-1β, TNF-α expression is increased [24]. However, during KOA progression, whether l-carnitine exerts pro-resolving effects of lipid mediators and safeguards effects of energy homeostasis by remodeling FLS development remains to be investigated.

In this study, we declared that l-carnitine supplementation can alleviate synovitis in knee osteoarthritis by regulating lipid accumulation and mitochondrial function. Further mechanistic studies revealed that l-carnitine ameliorates synovitis via the regulation of the AMPK-ACC-CPT1 signaling pathway. In conclusion, our study provides a rationale for the identification of l-carnitine as a potent therapeutic agent for the treatment of synovitis of KOA.

## Materials and methods

### Reagents

l-carnitine was obtained from Absin (Shanghai, China). Metformin was purchased from RHAWN (Shanghai, China). Compound C was obtained from APExBIO (Houston, USA). Etomoxir and HY-100965 were obtained from MedChemExpress (Monmouth Junction, NJ, USA). CCK8, D-Hanks and Rhodamine 123 were purchased from Solarbio (Beijing, China). LPS and 2′,7′-dichlorofluorescein diacetate were purchased from Sigma‒Aldrich (St. Louis, MO, USA). HE, Masson, Sirius Red, and TUNEL kits were purchased from Servicebio (Wuhan, China). Antibodies against AMPK (Cat No: 5831) and pAMPK (Cat No: 50081) were obtained from Cell Signaling Technology (Beverly, MA, USA). Primary antibodies, including anti-ACC (Cat No: AF6421), anti-pACC (Cat No: AF3421), anti-IL-1β (Cat No: AF5103), and anti-TNF-α (Cat No: AF7014) were supplied by Affinity Biosciences (Cincinnati, OH, USA). Antibodies for CPT1A (Cat No: 15184-1-AP) and TRPA1 (Cat No: 19124–1-AP) were purchased from Proteintech Group (Rosemont, IL, USA). Anti-cytochrome c (Cat No: 21680) was obtained from Signalway Antibody (Maryland, USA). FBS was obtained from TransGen Biotech (Beijing, China). 5 × HiScript II qRT SuperMix (Reverse transcription reagent) and TRIzol were obtained from Vazyme (Nanjing, China). Hieff qPCR SYBR Green Master Mix (Low Rox Plus) was obtained from Yeasen (Shanghai, China). DMEM was purchased from Nanjing BioChannel Biotechnology Co., Ltd. (Nanjing, China). RIPA, type I collagenase and serum medium Opti-MEM were obtained from Gibco (Rockville, USA). CultureSure Freezing Medium was supplied by FUJIFILM Wako Pure Chemical Corporation (Guangzhou, China). NcmECL Ultra was purchased from New Cell & Molecular Biotech (Suzhou, China). ELISA kits for IL-1β, IL-6, IL-8, IL-10, and TNF-α were supplied by Jinyibai Biotechnology Co., Ltd. (Nanjing, China). MDA, SOD, and CPT1 detection kits were supplied by Ziker Biological Technology Co., Ltd. (Shenzhen, Guangzhou, China). Enhanced ATP Assay Kit, Fluo-4 AM, Antifade Mounting Medium with DAPI, and BCA protein detection kit were supplied by Beyotime (Shanghai, China).

### Strategies for FLS

Primary FLS were isolated from male Sprague Dawley (SD) rats weighing approximately 180–200 g as described previously [25]. In brief, the synovial tissues were collected, washed with PBS, chopped with scissors, and soaked in PBS mix with type I collagenase (4 mg/mL) in cell culture incubators for 3 h. After filtration, centrifugation, precipitation, and resuspension, FLS were cultured in complete culture media (DMEM with 10% fetal bovine serum, 1% antibiotics) and maintained at 37 °C in a humidified incubator of 5% CO_2_ and 95% air. The morphology of FLS was assessed by an inverted microscope with a Leica Qwin System (Leica, Germany). To prevent deprivation of the cell phenotype, we chose FLS within the 3^rd^ to 6^th^ generations for successive experiments.

To explore the protective influences of l-carnitine, the cell processing protocol is as follows:

1. Control group: FLS were not treated except for medium replacement.
2. LPS group: FLS were exposed to 10 μg/mL LPS for 6 h.
3. LPS + l-carnitine: l-carnitine (2.5 mM) was administered for 24 h to LPS-treated FLS.
4. LPS + l-carnitine + Compound C: Compound C pretreatment at 10 μM for 12 h in LPS-treated FLS, and 2.5 mM l-carnitine was administered for 24 h.
5. LPS + l-carnitine + siRNA: siRNA pretreatment at 100 nM for 6 h in LPS-treated FLS, and 2.5 mM l-carnitine was administered for 24 h.

### Cell viability

Cell viability was determined by the CCK8 method according to the manufacturer’s protocol. Briefly, FLS were transferred to 96-well plates (10,000/well) for 24 h and then incubated in various concentrations of l-carnitine (2.5, 5, 10, and 100 mM) or DMSO alone for 24 h. Afterward, 10 μL of CCK8 was added to each well and incubated at 37 °C for 2 ~ 3 h. Finally, a microplate spectrophotometer (Envision, PerkinElmer, Waltham, MA) was used to detect the OD values at 450 nm.

### Transfection with small interfering RNA (siRNA)

Transfection with siRNA was performed according to our previous report [26]. Briefly, FLS were transfected with 100 nM AMPK siRNA (Genepharma, Shanghai, China), 100 nM CPT1 siRNA, or scrambled siRNA (sc-156134 and sc-37007; Santa Cruz, CA, USA) using Lipofectamine 2000 Transfection Reagent (Invitrogen, Carlsbad, CA, USA) with reduced serum medium Opti-MEM. The mRNA and protein levels of AMPK were measured by immunoblot and real-time PCR analyses. AMPK sequence: siRNA1: 5′-GCACGAGUUGACUGGACAUTT, AUGUCCAGUCAACUCGUGCTT-3′, siRNA2: 5′-CCUUUCUGGUGUGGACUAUTT, AUAGUCCACACCAGAAAGGTT-3′, siRNA3: 5′-CCAUUCUUGGUUGCCGAAATT, UUUCGGCAACCAAGAAUGGTT-3′. After 6 h of transfection, the solution was exhausted and incubated in a cell culture medium for 24 h to validate the transfection efficiency.

### Biochemical measurements

After drug administration treatment, cells or tissues were collected and crushed using an ultrasonic cell disrupter system (Sonopuls, Enraf Nonius, Rotterdam, The Netherlands). Carnitine palmitoyltransferase-1 (CPT1), superoxide dismutase (SOD), and malonaldehyde (MDA) were detected using the corresponding assay kits. The experimental procedures strictly followed the instructions of the manufacturer.

### ATP Production, ΔΨm and ROS

ATP content was determined using the Enhanced ATP Assay Kit. Lysed cells or tissues were centrifuged at 12,000×*g* for 10 min, and the supernatant was collected. FLS were lysed with 200 μL of ATP-lysate reagent and centrifuged at 12,000×*g* for 10 min. Subsequently, 20 μL of the supernatant was transferred into a 96-well black lightproof plate, and then 100 μL of ATP detection reagent was added to each well for 5 min. The chemiluminescence of each well was monitored on a microplate spectrophotometer (Envision, PerkinElmer, Waltham, MA) in luminance mode within 30 min.

The ΔΨm was measured using Rhodamine 123. FLS were rinsed with PBS twice, and 2 mL of Rhodamine 123 solution (5 μm) was added to each well and dyed at 37 °C for 20 min in the dark. The staining solution was discarded, and the FLS were washed 3 times with D-Hanks. The change in ΔΨm was observed under a Nikon Eclipse E100 (Nikon, Japan), and the fluorescence intensity reflected the level of ΔΨm.

ROS levels were detected using 2′,7′-dichlorofluorescein diacetate according to the manufacturer's protocol. ROS working solution (10 μm) was added to FLS and incubated at 37 °C for 15 min in the dark. Then, the cells were washed with D-Hanks twice and photographed under a Nikon Eclipse E100 (Nikon, Japan). The average fluorescence intensity of the cells was analyzed using Image-Pro Plus software (Media Cybernetics Inc.).

### Immunofluorescence and Confocal Microscopy

Fluorescence staining was performed as described previously [26]. Briefly, FLS were seeded on glass slides, washed twice with D-Hanks, fixed with 4% paraformaldehyde, and then permeabilized with 0.2% Triton X-100. Then, 5% BSA was employed to block nonspecific antigens on each slide. Thereafter, D-Hanks was used to rinse the samples, which were inoculated with primary antibodies against pAMPK (1:300) and CPT1 (1:300) at cold storage overnight. Rinsing of glass plates was done the next day and then inoculated with CoraLite594 or Fluor488-conjugated secondary antibodies (1:200) in the dark for 1 h, followed by labeling using Antifade Mounting Medium with DAPI for 10 min. A random selection of six regions for every slide was observed and captured under an LSM700 laser scanning confocal microscope (Zeiss, Germany).

### ***Ca***^***2***+^***-influx Measurements***

Calcium influx in FLS after TRPA1 opening was observed by calcium imaging as in our previous study [24]. In brief, FLS were incubated with 5 μM Fluo-4-AM for 30 min, and the intracellular influx of calcium was observed under an LSM700 laser scanning confocal microscope (Zeiss, Germany) at a 1/s frequency. Then, FLS were washed twice with D-Hanks to eliminate extracellular Fluo-4-AM. In the measurements, basal fluorescence was first recorded for 30 s until the TRPA1 agonist (HY-100965, 100 μM) was added at 37 °C, and the following measurement ensued for 60 s.

### Western blotting

Western blot analysis was performed as described previously [27]. In short, tissue or cellular proteins were extracted using RIPA buffer containing 0.1% PMSF and then quantified by the BCA method. Aliquots of protein lysates were electrophoresed on a sodium dodecyl sulfate–polyacrylamide gel, transferred from the gel onto a PVDF membrane, and blocked with 5% BSA for 1 h. Membranes were incubated overnight at 4 °C with a range of 1:1000–2000 dilutions of AMPK, pAMPK, ACC, pACC, TRPA1, IL-1β, TNF-α, cyc, and β-actin primary antibodies, followed by HRP-conjugated secondary antibodies. Protein bands were visualized with an ECL detection kit, and the intensity of protein bands was quantitated by Image Lab 6.1 software (Bio-Rad Laboratories, Hercules, CA).

### Quantitative Real-time PCR

RNA extraction and quantitative real-time PCR were performed according to our previously reported method [27]. Briefly, total RNA was extracted from synovial tissues and FLS using TRIzol. Then, researchers estimated the contamination by the A260/280 (between 1.8 and 2.0) and the integrity by electrophoresis to confirm RNA quality, which includes purity and integrity. Next, cDNA was synthesized using 1 µg of RNA with a PrimeScript RT Reagent Kit with gDNA Eraser following a set procedure (50 °C, 15 min; 85 °C, 5 s). PCRs were performed using qPCR mix according to the manufacturer's instructions in an ABI 7500 PCR system (Applied Biosystems, USA). The gene expression level was normalized to that of the external control housekeeping gene β-actin mRNA and calculated by the 2^−ΔΔCT^ data analysis method. The primer sequences are given in Table 1.

**Table 1:** Primer sequences of the target genes

Gene	Forward	Reverse
*AMPK* *ACC* *TRPA1* *IL-1β* *TNF-α* *CPT1A* *β-actin*	AGATATCAGGGAACATGAATGG GAGTCTGGCTACTACTTGGA GAATTTCCAAGATGCCTTCAG TTCATCTTTGAAGAAGAGCCC CTTCTCATTCCTGCTCGTG ATTTGTGGGAGTATGTCATGG CTTCCTGGGTATGGAATCCT	ATGGTTGAACTATAAGACGGG ATACGCCTGAAACATGATCTG CGGTAATTGATGTCTCCCAG CTGTCTAATGGGAACATCACAC TTTGGGAACTTCTCCTCCT GGTTGGTGTCTCCTTTACAG TCTTTACGGATGTCAACGTC

### ELISA

The inflammatory cytokines in the supernatants of FLS or the serum of rats were detected with ELISA kits as described by the manufacturer. The absorbance was measured at 450 nm using a microplate spectrophotometer (Envision, PerkinElmer, Waltham, MA).

### Animals

Four-week-old male SD rats of specific pathogen-free (SPF) grade for experiments were obtained from Nanjing Qinglong-shan Animal Farm (SCXK(SU) 2017-0001, China). The animals were kept adaptively for 7 days. Rats were housed in an SPF-grade environment with controlled temperature and humidity and were free of specific pathogens. The protocol for animal care and usage was approved by the Animal Care and Use Committee of Nanjing University of Chinese Medicine (Approval number: A210101) and followed the Guide for the Care and Use of Laboratory Animals of the National Institutes of Health.

Animals were randomly numbered and divided into five groups (n = 6 in each group): control group, ACLT group, l-carnitine group, l-carnitine + Metformin group, and l-carnitine + Etomoxir group. Figure 4a shows the overall timeline of our animal experiments. After one week of adaptive feeding, we started modeling, and that day was recorded as Day 7. The rats in the control group were subjected to a sham operation and were later treated with 0.9% NaCl only. Except for the control group, the anterior cruciate ligament (ACLT) was destroyed in the other four groups of rats, both knees. On Day 21, rats in the l-carnitine group received l-carnitine orally at a concentration of 100 mg/kg of rat weight, once a day, for 4 weeks [22]. In addition to oral l-carnitine, metformin was administered either intraperitoneally (ip, solubilized in 0.9% NaCl) at a concentration of 100 mg/kg of rat weight twice a day for 4 weeks in the l-carnitine + Metformin group [28]. In addition to oral l-carnitine, etomoxir (5 mg/kg of body weight) was dissolved in 0.9% (w/v) NaCl and administered intraperitoneally once every three days for 4 weeks in the l-carnitine + Etomoxir group [19]. At the end of the administration, we sacrificed the rats by intraperitoneal injection of 3% pentobarbital sodium (100 mg/kg), and the tissue samples were processed differently according to the different experimental protocols.

### Histological analysis

Synovial tissues were fixed in 4% paraformaldehyde, paraffin-embedded, and then transversely sectioned for routine HE staining. Sirius Red and Masson staining were performed according to the instructions of the kit. Approximately 5 μm samples were observed under a Leica DMI-3000B microscope (Leica, Germany). The histological score of the synovium was determined according to Krenn’s score [29]. The degree of synovial fibrosis was evaluated by calculating the positive area of collagen fibers and the percentage of collagen I positive areas with ImageJ (National Institutes of Health, USA, available at http://rsbweb.nih.gov/ij/).

### TUNEL staining

The degree of apoptosis was detected by TUNEL staining in synovial tissues. All experiments were performed according to the manufacturer’s instructions. Tissue sections were observed under a Leica DMI-3000B microscope (Leica, Germany). The level of apoptosis is shown as a percentage of the number of TUNEL-positive cells to the number of total cells.

### Immunohistochemistry

Paraffin sections of synovial tissues were deparaffinized, rehydrated in descending grades of ethanol, preincubated with BSA for 10 min, and then treated in cold storage overnight with primary antibody (CPT1; 1:500 dilution). The sections were HRP-conjugated IgG secondary antibodies (1:1000 dilution) for 30 min. Finally, the slices were rinsed with PBS, and images of the sample sections were acquired using a Leica DMI-3000B microscope (Leica, Germany). The positive cells were quantified using ImageJ software.

### Statistical analysis

The experimental data are presented as mean ± SD. GraphPad Prism 7.14 Software (San Diego, CA, USA) was employed to analyze the data and prepare graphs. Data were assessed with one-way analysis of variance (ANOVA) and then by Tukey's test for comparison between model and treatment groups. A value of *P* < 0.05 was considered statistically significant.

## Results

### AMPK mediated the synovioprotective effect of l-carnitine in an LPS-stimulated FLS model

**Fig. 1:**
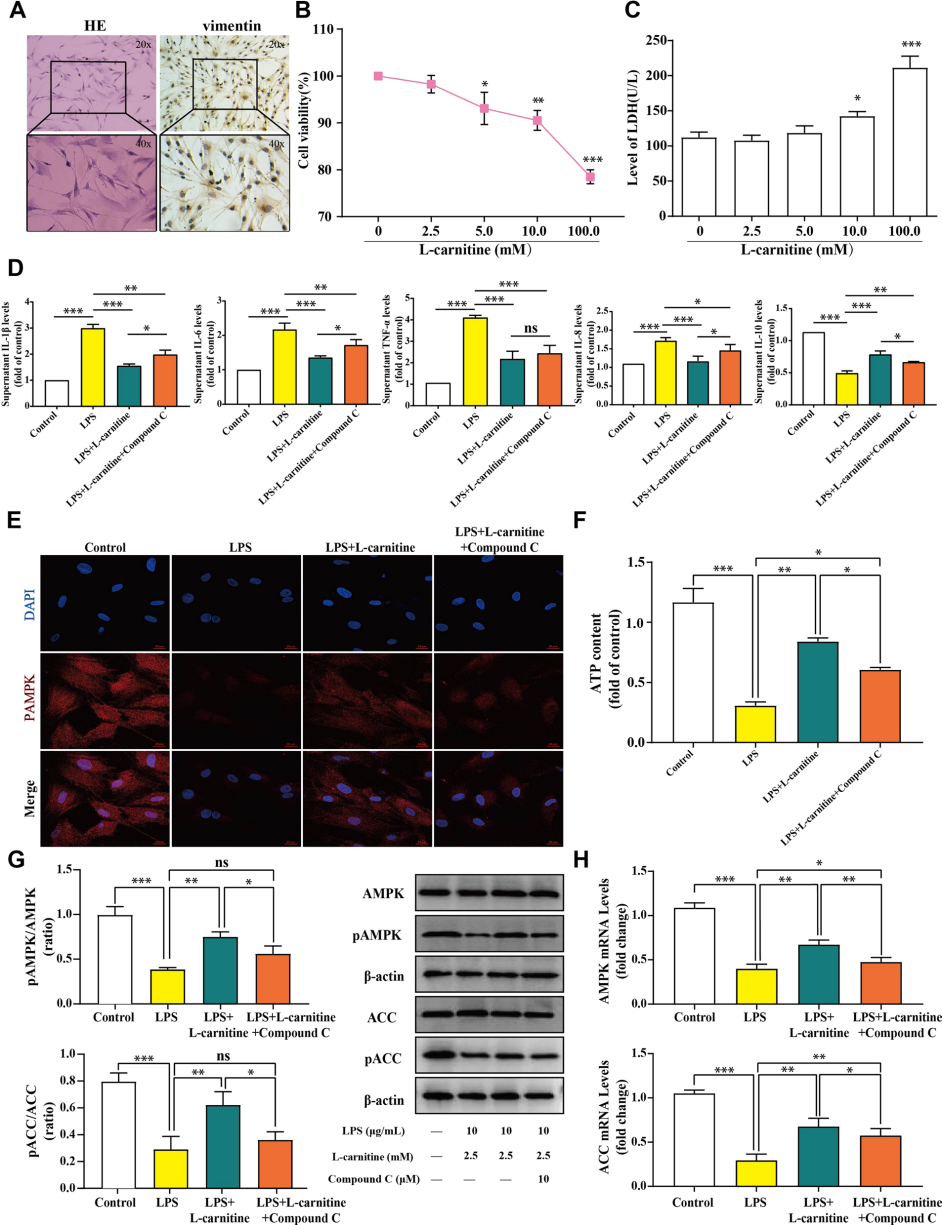
AMPK mediated the synovioprotective effect of l-carnitine in an LPS-stimulated FLS model. **a** HE and anti-vimentin immunostaining of FLS for cell identification. **b** The cell viability of l-carnitine on FLS. **c** Toxicity was detected by LDH. **d** ELISA measurement of IL-1β, IL-6, IL-8, TNF-α, and IL-10 levels in the supernatant. **e** Immunostaining shows that pAMPK is increased after l-carnitine administration (× 630 magnification). **f** ATP detection shows that l-carnitine can replenish ATP levels. **g** Immunoblot analysis of AMPK, pAMPK, ACC, and pACC. **h** Real-time PCR data of AMPK and ACC. Data are presented as the mean ± SD (n = 3). ns, not significant. *P < 0.05; **P < 0.01; ***P < 0.001

Our previous data have sufficiently demonstrated that FLS are involved in the progression of KOA via the secretion of various proinflammatory cytokines, such as IL-1β, IL-6, IL-8, and TNF-α [27, 30]. To investigate the mechanisms underlying the protective effects of l-carnitine, we proposed that l-carnitine may protect against LPS-induced inflammation. FLS were first identified by HE and anti-vimentin immunostaining (Fig. 1a). The CCK8 assay was performed to investigate the viability of FLS treated with different l-carnitine concentrations (0, 2.5, 5, 10, and 100 mM) (Fig. 1b). Meanwhile, the LDH assay revealed that l-carnitine has no side toxicity to FLS at concentrations lower than 10 mM (Fig. 1c). To further verify the role of AMPK in the anti-inflammatory effect, we used the AMPK inhibitor compound C. Compound C was preincubated with FLS for 12 h to inhibit the activation of AMPK in advance, and FLS were then treated with l-carnitine for another 24 h. Cell supernatant was collected from each group, and the levels of IL-1β, IL-6, TNF-α, IL-8, and IL-10 were determined by ELISA. As shown in Fig. 1d, l-carnitine not only inhibited the expression of proinflammatory factors (IL-1β, IL-6, TNF-α, and IL-8) but also promoted the production of an anti-inflammatory factor (IL-10), suggesting the anti-inflammatory properties of l-carnitine. In addition, to investigate whether the regulation of AMPK/ACC by l-carnitine could play a pivotal role in the anti-inflammatory effects, we performed immunofluorescence (Fig. 1e), ATP detection (Fig. 1f), immunoblot analysis (Fig. 1g), and real-time PCR (Fig. 1h). As expected, the contents of pAMPK and pACC in the l-carnitine group were significantly increased compared with those in the LPS group, and l-carnitine increased the level of ATP. Moreover, compound C preadministration eliminated the enhanced activity of l-carnitine on AMPK/ACC signaling. Taken together, these findings suggest that l-carnitine is a potent drug that increases the phosphorylation of AMPK and ACC to reduce synovial inflammation in the LPS-stimulated FLS model.

### l-carnitine induced CPT1 upregulation mediated by AMPK in an LPS-stimulated FLS model

**Fig. 2:**
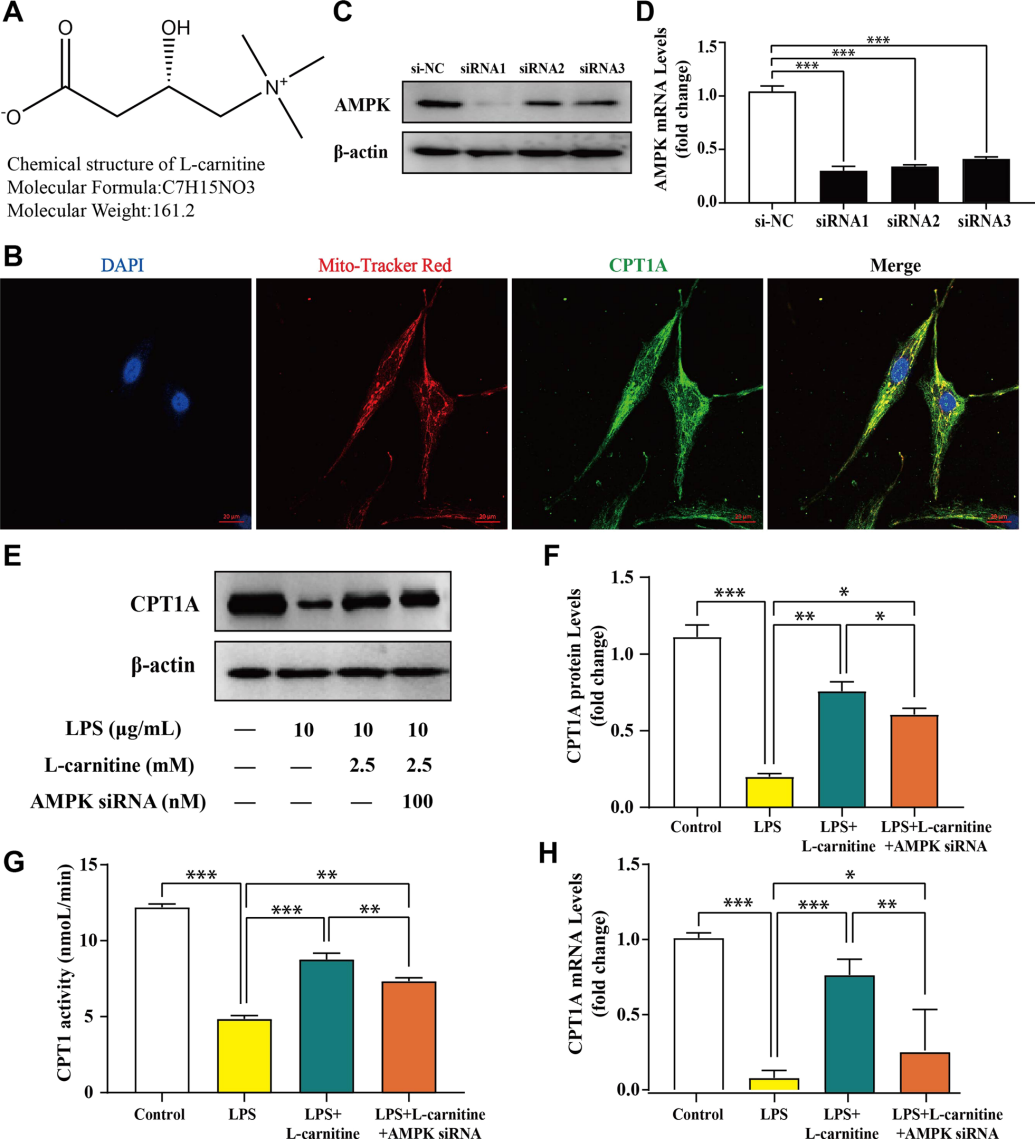
l-carnitine induced CPT1 upregulation mediated by AMPK in an LPS-stimulated FLS model. **a** The chemical structural formula of l-carnitine. **b** Immunostaining shows that CPT1A is located in the mitochondrion (× 630 magnification). **c, d** The silencing effect of the AMPK siRNA was confirmed by immunoblot and real-time PCR analyses. **e, f** Immunoblot analysis showing the effects of l-carnitine (2.5 mM) or AMPK siRNA (100 nM) on CPT1 in primary FLS exposed LPS. **g** The l-carnitine group showed a significant upregulation of CPT1 activity compared with the LPS group. **h** PCR data showing the mRNA levels of CPT1 in FLS treated with 2.5 mM l-carnitine for 24 h compared to LPS-treated cells. Data are presented as the mean ± SD (n = 3). *P < 0.05; **P < 0.01; ***P < 0.001

l-carnitine, as a substrate of CPT1, is a naturally occurring bioactive amino acid that promotes the conversion of fat into energy and therefore has an important role in lipid lowering (Fig. 2a). CPT1A is known to be expressed in the liver, lymphocytes, and fibroblasts [18], but its expression and location in FLS are unknown. Further experiments demonstrated that CPT1A is mainly located in the mitochondria of living cells, which can be directly and conveniently observed by confocal microscopy (Fig. 2b). Numerous studies have shown a crucial role for the AMPK/CPT1 pathway in inflammatory diseases [31]. In this study, we hypothesized that l-carnitine induced CPT1 upregulation mediated by AMPK in the LPS-stimulated FLS model. To test this hypothesis, we employed AMPK siRNA to investigate the effect of l-carnitine on the AMPK/CPT1 pathway in FLS (Fig. 2c, d). Next, several assessment factors of CPT1 were detected by immunoblot and densitometric analysis (Fig. 2e, f), CPT1 activity analysis (Fig. 2g), and real-time PCR analysis (Fig. 2h) in LPS-treated FLS. The obtained results revealed that inhibition of AMPK expression led to a decrease in the level of CPT1 and that l-carnitine could increase the level of CPT1. Overall, these data suggest that CPT1 is a downstream protein of AMPK and that l-carnitine treatment increases CPT1 expression in the FLS model.

### l-carnitine regulates lipid accumulation and mitochondrial function and inhibits TRPA1-mediated inflammatory amplification through the CPT1 signaling pathway in vitro

**Fig. 3:**
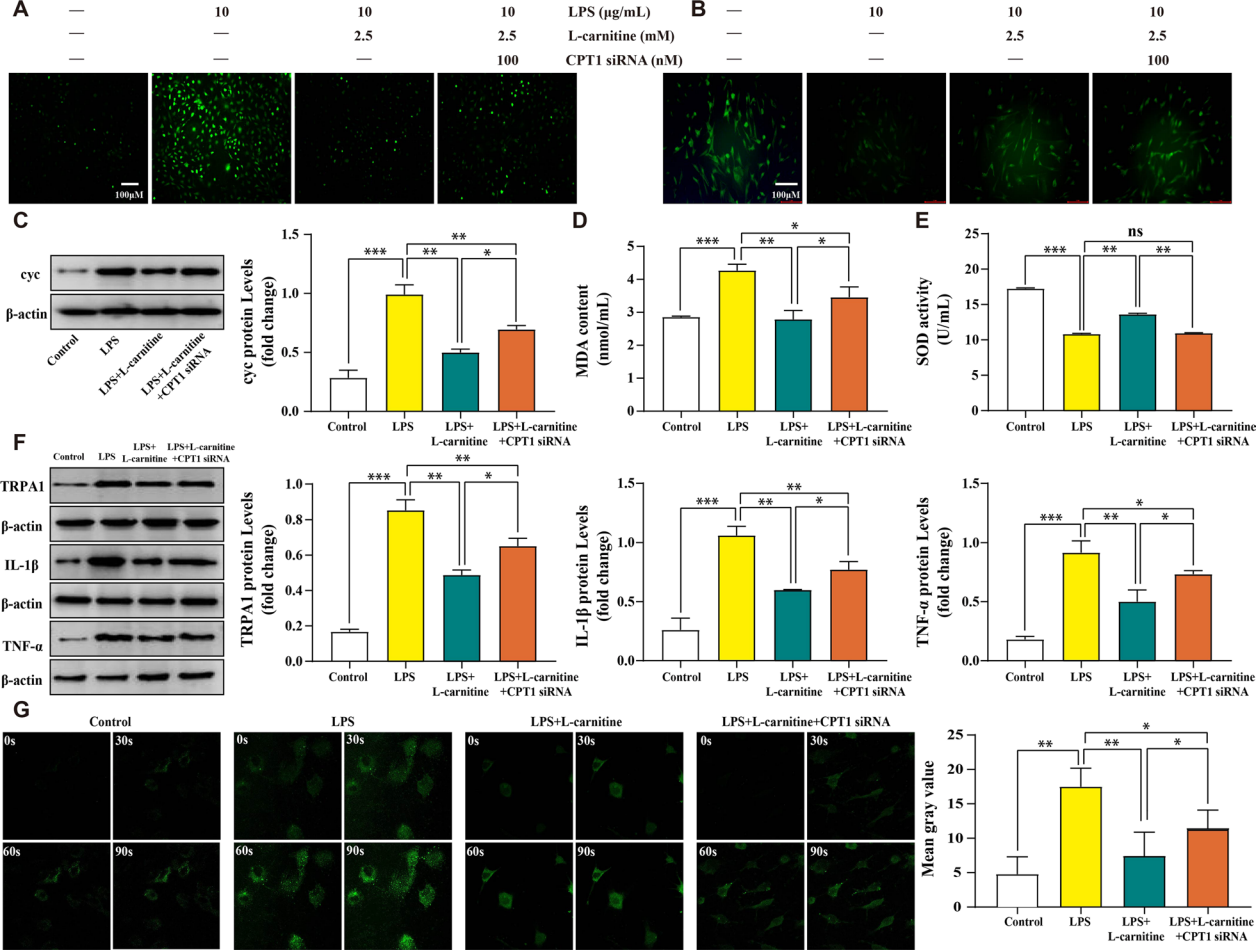
l-carnitine regulates lipid accumulation and mitochondrial function and inhibits TRPA1-mediated inflammatory amplification through the CPT1 signaling pathway in vitro. **a, b** l-carnitine administration significantly reduced the production of ROS (× 100 magnification) and preserved ΔΨm (× 20 magnification). **c** The protein levels of cyc were determined. **d, e** l-carnitine administration significantly reduced the content of MDA and increased the antioxidant enzymes (SOD activity). **f** Immunoblot analysis of TRPA1, IL-1β and TNF-α. Representative blots were from three independent experiments. **g** Representative photographs of Ca.^2+^ fluorescence intensity in different FLS treatment groups at 0, 30, 60, and 90 s (× 630 magnification). Data are presented as the mean ± SD (n = 3). ns, not significant. *P < 0.05; **P < 0.01; ***P < 0.001

To determine whether l-carnitine regulates lipid accumulation and mitochondrial function and inhibits TRPA1-mediated inflammatory amplification through the CPT1 signaling pathway in FLS, we used various biological methods to examine the effect of l-carnitine. First, CPT1 siRNA was used in this experiment, and we found that treatment with 2.5 mM l-carnitine significantly decreased the cellular ROS content (Fig. 3a). Cell apoptosis leads to a decrease in ΔΨm and a reduction in the fluorescence intensity in FLS. Following Rhodamine 123 staining, the obtained results showed that the LPS group exhibited a loss in ΔΨm and that l-carnitine significantly enhanced the fluorescence intensity of ΔΨm (Fig. 3b). Cytochrome c (Cyc) is released from the mitochondrial inner membrane into the cytoplasm when mitochondria are disrupted. l-carnitine administration significantly decreased the level of cellular cyc compared with LPS-treated FLS (Fig. 3c). Second, lipid accumulation was partially assessed by MDA content and SOD activity. We observed a decrease in MDA content in FLS cultured with l-carnitine for 24 h, but the reverse pattern of SOD activity was observed (Fig. 3d, e). Third, protein detection experiments demonstrated that the elevated expression of TRPA1, IL-1β, and TNF-α in LPS-treated FLS was abolished by treatment with l-carnitine (Fig. 3f). Last, our previous studies confirmed that LPS enhanced Ca^2+^ influx, as mediated by TRPA1 in OA-FLS [24]. We also investigated whether the anti-inflammatory effect of l-carnitine is related to the TRPA1-mediated LPS-induced inflammatory response in FLS. The obtained results showed that l-carnitine inhibited TRPA1-mediated calcium influx (Fig. 3g). Collectively, these data demonstrate that l-carnitine can regulate lipid accumulation and mitochondrial function and inhibit TRPA1-mediated inflammatory amplification through the CPT1 signaling pathway.

### l-carnitine induced AMPK phosphorylation and expression to protect against synovitis and delayed OA progression in the ACLT-induced model

**Fig. 4:**
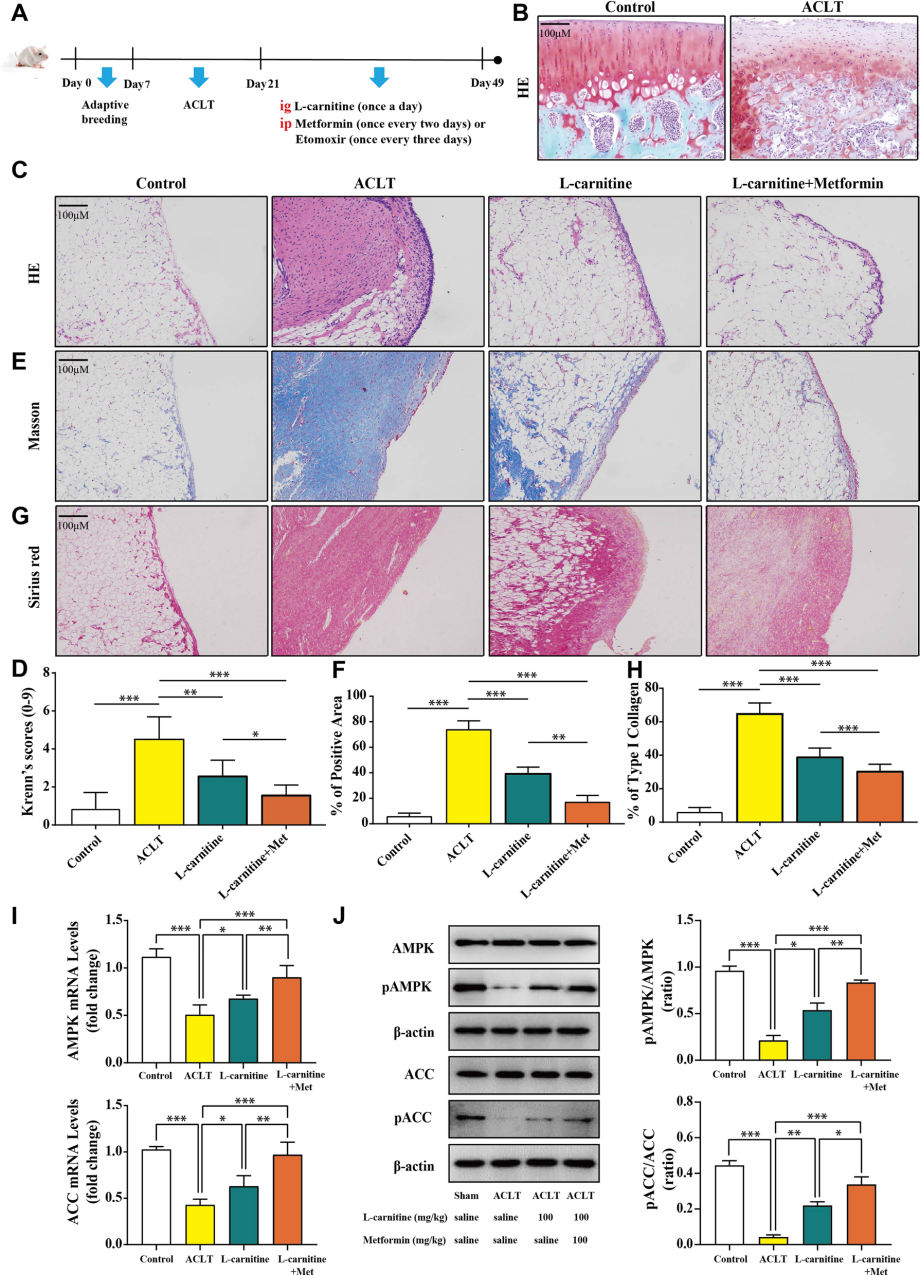
AMPK phosphorylation and expression protect against synovitis and delay OA progression in the ACLT-induced model. **a** Process diagram of animal experiment. **b** HE staining of cartilage (× 100 magnification). **c** HE staining of synovial tissue (× 100 magnification, n = 6). **d** Krenn’s scores of synovitis in each group. **e** Masson staining of synovial tissue (× 100 magnification, n = 6). **f** Masson’s trichrome-positive area in each group. **g** Sirius Red staining of synovial tissue (× 100 magnification, n = 6). **h** Type I collagen (% Area) in each group. **i** Real-time PCR data of AMPK and ACC (n = 3). **j** Immunoblot analysis of AMPK, pAMPK, ACC, and pACC (n = 3). Data are presented as the mean ± SD. *P < 0.05; **P < 0.01; ***P < 0.001

The results of the dissociative experiments prompted us to further investigate the anti-inflammatory potential of l-carnitine in animal experiments. The animal experiment process is shown in Fig. 4a, and the KOA model was identified by HE staining of cartilage (Fig. 4b). To directly assess whether l-carnitine could induce a histopathologically ameliorative effect on synovitis, HE, Masson, and Sirius Red staining were applied. HE staining (Fig. 4c) showed that the ACLT group exhibited a large infiltration of inflammatory cells, disorganized synovial cell arrangement, and increased density and proliferation of synovial cells. The rats that received l-carnitine alone or in combination with metformin exhibited less inflammatory cell infiltration, resident cell hyperplasia, and formation of lining cell layers than the ACLT group. Krenn’s scores were consistent with synovial pathology (Fig. 4d). Masson staining (Fig. 4e, f) showed that the l-carnitine and l-carnitine + Metformin groups had less synovial fibrosis than the ACLT group. Moreover, Sirius Red staining showed that the l-carnitine groups had decreased deposition of type I collagen fibers compared to the ACLT group (Fig. 4g, h). Meanwhile, regardless of HE, Masson, or Sirius Red staining, the l-carnitine + Metformin groups had a better alleviating effect on inflammation and fibrosis than the l-carnitine group. The results of real-time PCR (Fig. 4i) and immunoblot analysis (Fig. 4j), which were consistent with the cell experiments, showed that l-carnitine increased the levels of pAMPK and pACC. Taken together, these data show that l-carnitine can limit synovitis in ACLT-induced KOA animals and that its mechanisms may be associated with the increased phosphorylation of AMPK and ACC.

### l-carnitine-induced enhancement of CPT1 mediated by AMPK protects against synovitis in the synovial tissue of rats

**Fig. 5:**
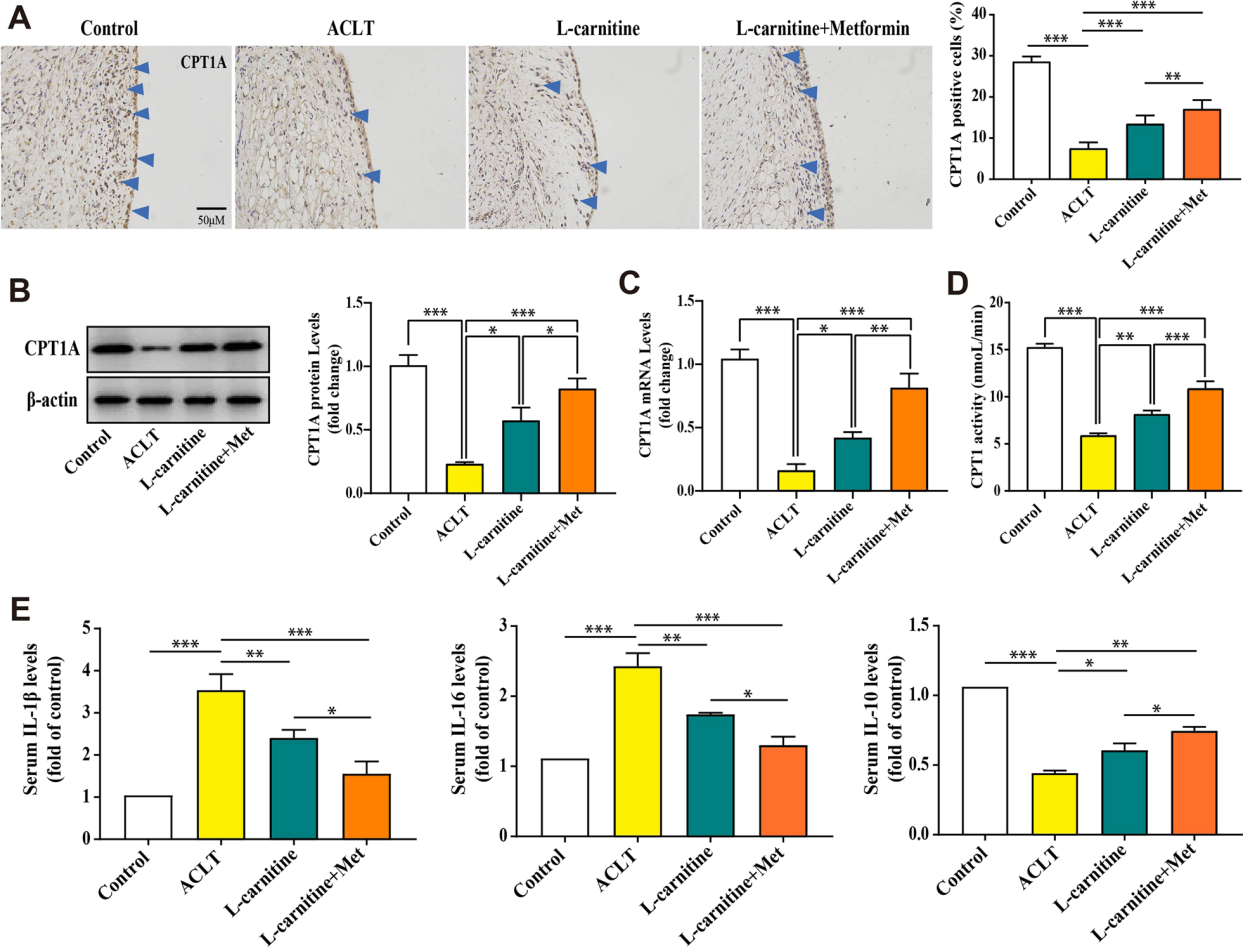
l-carnitine-induced enhancement of CPT1 mediated by AMPK protects against synovitis in the synovial tissue of rats. **a** Immunohistochemistry shows that the l-carnitine group showed a significant upregulation of CPT1 compared with the ACLT group (positive cells are shown with blue arrows, × 200 magnification, n = 6). **b** Immunoblot and densitometric analysis of CPT1 (n = 3). **c** Real-time PCR analysis of CPT1 (n = 3). **d** CPT1 activity analysis in the serum (n = 3). **e** ELISA measurement of IL-1β, IL-6, and IL-10 levels in the serum (n = 6). Data are presented as the mean ± SD. *P < 0.05; **P < 0.01; ***P < 0.001

Numerous studies have shown that CPT1 catalyzes the reversible transfer of acyl groups between coenzyme A (CoA) and l-carnitine [17]. In this study, we hypothesized that l-carnitine induced an enhancement of AMPK-mediated CPT1 that may play a key role in the protection against synovitis of KOA. To test this hypothesis, we used different biological methods to examine the effect of l-carnitine. First, immunohistochemistry results revealed that l-carnitine treatment clearly increased the level of CPT1 (Fig. 5a). In addition, several assessment factors of CPT1 were detected by immunoblot, and densitometric analysis (Fig. 5b), real-time PCR analysis (Fig. 5c), and CPT1 activity analysis (Fig. 5d) in the ACLT model. The obtained results revealed that l-carnitine increased the level of CPT1. Finally, ELISA of rat serum demonstrated that l-carnitine reduced the expression of inflammatory cytokines, including IL-1β and IL-6, and promoted the production of anti-inflammatory factors, including IL-10 (Fig. 5e). Overall, these data suggest that overexpression of AMPK increases CPT1 expression levels, and l-carnitine administration increases CPT1 expression in the ACLT-induced KOA model.

### l-carnitine improved mitochondrial function and lipid accumulation through CPT1 to achieve anti-inflammatory effects in the KOA model of rat

**Fig. 6:**
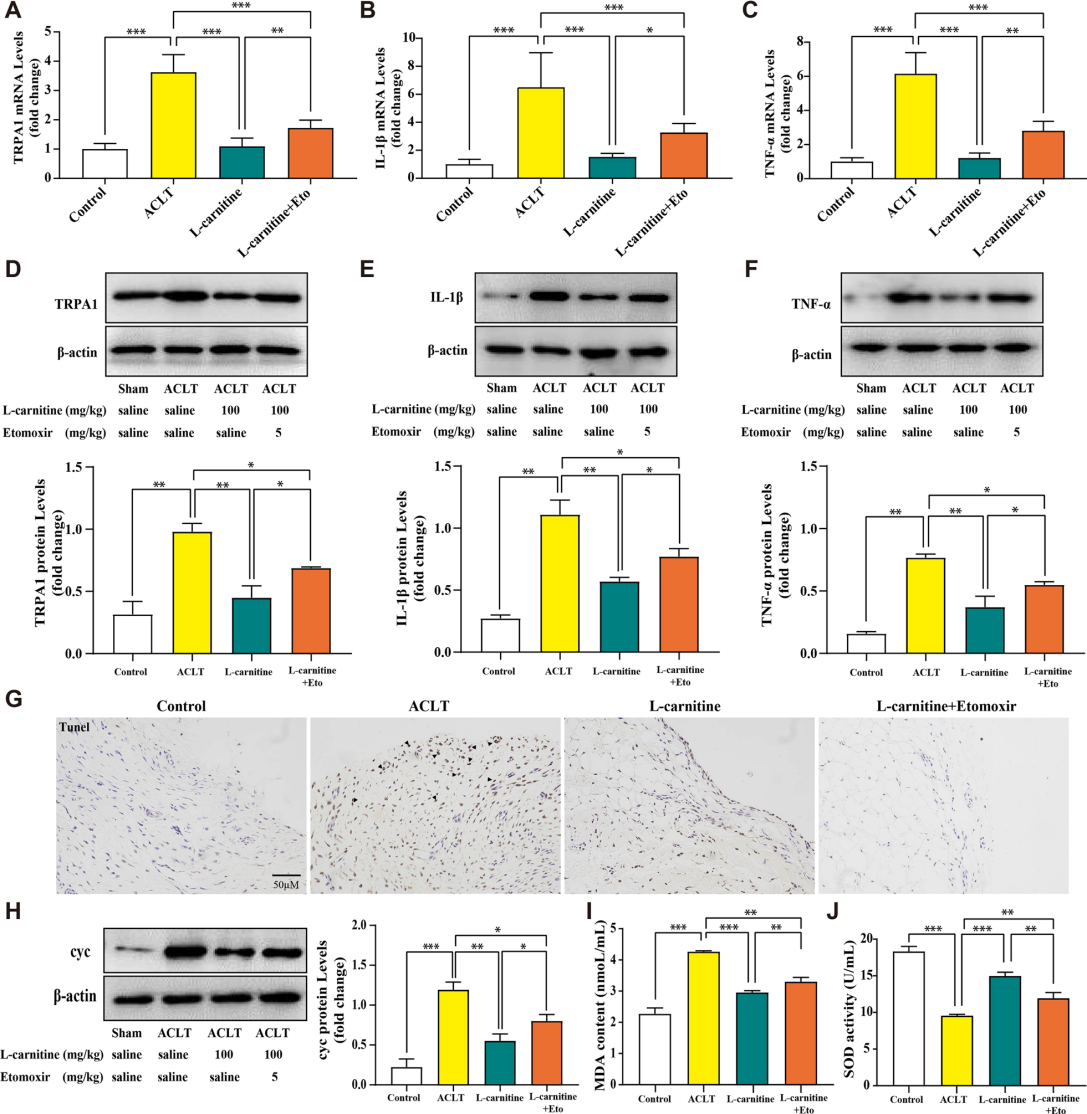
l-carnitine improved mitochondrial function and lipid accumulation through CPT1 to achieve anti-inflammatory effects in the KOA model of rats. **a–c** Real-time PCR data of TRPA1, IL-1β, and TNF-α. **d–f** Immunoblot analysis of TRPA1, IL-1β, and TNF-α. **g** TUNEL-positive cells are shown in KOA synovia (black arrows, × 200 magnification). **h** Immunoblot analysis of cyc was performed. **i, j** Detection of MDA content and SOD activity. Data are presented as the mean ± SD (n = 3). *P < 0.05; **P < 0.01; ***P < 0.001

Finally, to confirm whether l-carnitine improved mitochondrial function and lipid accumulation through CPT1 to achieve anti-inflammatory effects in the ACLT-induced KOA model, we used different biological methods to examine the effect of l-carnitine. First, etomoxir, a potent irreversible inhibitor of CPT1, was used in this experiment, and some research data have shown that it can inhibit lipid oxidation by targeting CPT1 [32]. Then, we examined inflammation-related proteins and genes in the ACLT-induced KOA model by real-time PCR analysis (Fig. 6a–c), immunoblot and densitometric analysis (Fig. 6d–f). The obtained results showed the significant inhibition of protein and mRNA expression of TRPA1, IL-1β, and TNF-α by treatment with l-carnitine in comparison to the ACLT group. Next, we analyzed mitochondrial function by detecting TUNEL staining and cyc expression. We found that the number of TUNEL-positive cells increased in the ACLT group, while l-carnitine inhibited this upregulation (Fig. 6g). In addition, the level of cyc was significantly downregulated by treatment with l-carnitine (Fig. 6h). Moreover, MDA content and SOD activity in serum were applied to evaluate lipid accumulation. l-carnitine not only inhibited the content of MDA but also promoted the production of SOD activity (Fig. 6i, j). Collectively, these data demonstrate that l-carnitine can regulate lipid accumulation and mitochondrial function and inhibit the inflammatory response via the CPT1 signaling pathway.

## Discussion

KOA is one of the most common disabling joint disorders, and approximately 44.7% of patients with OA are at risk of developing a disability in their lifetime [4]. This situation is not optimistic because the aging population is rapidly increasing. KOA is characterized by articular cartilage injury, accompanied by synovitis and subchondral bone sclerosis [5]. Major risk factors for KOA include obesity, age, genetics, trauma, and metabolism [2, 3]. As one of the risk factors, obesity has a role in OA, including increased joint loads, altered metabolism, and low-grade inflammation [33]. Obesity can cause several metabolic abnormalities, one of which is abnormal lipid metabolism [34]. Therefore, it is worth evaluating the direct effects of metabolic pathways on KOA, especially in circumstances of obesity and energy deficiency. To date, there are no known therapies that can alter the progression of KOA or prevent its occurrence [6]. Thus, the focus of KOA management remains on alleviating inflammatory conditions [35].

Here, we have revealed and characterized a unique mechanism of l-carnitine-induced anti-inflammatory effects in KOA synovitis, and the underlying mechanism may be related to improving mitochondrial function and reducing lipid accumulation via the AMPK-ACC-CPT1 signaling pathway. This may serve as a potential target for KOA therapy. More particularly, we found that l-carnitine may protect the synovium against LPS-induced and ACLT-induced impairment by suppressing inflammation. Treatment with l-carnitine can upgrade the inflammatory microenvironment of KOA synovitis in vivo and inhibit the production and release of inflammatory cytokines in vitro. Consistent with a previous study, a potential mechanism for the anti-inflammatory effect of l-carnitine is related to the inhibition of TRPA1-mediated calcium influx. Interestingly, we found that CPT1 is a downstream protein of AMPK, and inhibition of AMPK expression leads to a decrease in the level of CPT1. Moreover, l-carnitine treatment increased pAMPK, pACC, and CPT1 expression in the FLS model and ACLT animal model. Importantly, we further found that l-carnitine has a critical role in alleviating synovitis in the KOA model by regulating lipid accumulation (ROS generation, MDA content, and SOD activity) and mitochondrial function (ROS generation, cellular ATP levels, ΔΨm, and cell apoptosis). To our knowledge, few studies have been reported to estimate the role of l-carnitine in protecting synovitis [22], in particular the protection against excessive inflammation via the AMPK-ACC-CPT1-dependent energy pathway is not seen.

AMPK is a critical bioenergy sensor, and the functional role of AMPK has been extensively studied in lipid metabolism (fatty acids, mitochondria, and cholesterol), glucose metabolism, protein synthesis, inflammation mitigation, mitochondrial biogenesis, apoptosis, and many other aspects [21]. AMPK is known to play a role in the treatment of obesity and metabolic disorders by inhibiting anabolic pathways and enhancing catabolic pathways [36]. Therefore, AMPK has become a promising therapeutic strategy for regulating the energy homeostasis of specific organs or tissues to some extent. Most of the research on AMPK in KOA focuses on cartilage, and some studies reveal the relationship between abnormal AMPK activity and synovial pathological changes in OA. For example, chitosan oligosaccharide ameliorated the degree of synovitis in vitro and in vivo via AMPK activation [37]. Moreover, AMPK plays a primary role in controlling lipid metabolism by modulating the downstream ACC and CPT1 pathways [36]. As demonstrated above, the activation of AMPK induces the inhibitory phosphorylation of ACC. ACC is a vital enzyme in fatty acid synthesis that can catalyze the carboxylation of acetyl-CoA to malonyl-CoA [17]. CPT1 is a mitochondrial importer of fatty acids; a previous study revealed that inhibition of CPT1 significantly increases the level of oxylipin during inflammation in vivo [38]. In fibroblasts, another study showed that CPT1 can reverse cellular senescence by regulating lipid accumulation and mitochondrial function [39]. Additionally, many studies have shown that l-carnitine can restore CPT1 activity [22, 40]. Our results were in accordance with these published works, and l-carnitine markedly increased CPT1 activity, similar to metformin [41]. Our outcomes reported that l-carnitine treatment significantly improved AMPK activity, inhibited ACC activity, and eventually increased CPT1A function.

The imbalance between lipid peroxidation systems and lipid antioxidation systems leads to the formation of oxidation products [42]. Overproduction of ROS and induction of oxidative stress are major contributors to OA pathogenesis [13]. An increase in ROS results in the activation of the cellular defense mechanism against lipid oxidation to eliminate ROS [38]. Defective or insufficient mitochondrial function at the cellular level is thought to impact whole-body metabolic homeostasis. The lipid antioxidation system includes various enzymes, such as superoxide dismutases (SOD) and glutathione peroxidase (GPx) [38]. SOD is an important endogenous antioxidant enzyme and free radical scavenger whose function is to maintain metabolic balance [43]. Thiobarbituric acid reactive substances (TBARS) are biomarkers of oxidative stress, the quantification of which reflects the MDA content. MDA content is an important parameter reflecting the body's potential antioxidant capacity, which can not only reflect the rate and intensity of lipid peroxidation but also indirectly reflect the degree of tissue peroxidation damage [44]. In our study, we used spectrophotometry and the microplate method to determine the activity of SOD and the MDA content. We found that l-carnitine promoted SOD activity and decreased the MDA content. On the other hand, we found that l-carnitine significantly decreased the content of cellular ROS and cyc protein and increased the level of ΔΨm. These findings indicated that l-carnitine plays a critical role in ameliorating peroxidative damage, restoring mitochondrial dysfunction, regulating the homeostasis of lipid metabolism, protecting cell function, and avoiding death. l-carnitine, as a prospective “fat burner,” is extensively employed as one of the most effective ways to promote endurance and fat combustion and shorten postworkout recovery [45, 46]. l-carnitine has a variety of roles in fatty acid metabolism, the most important of which is to participate in the classical “carnitine shuttle” process [20]. Specifically, l-carnitine facilitates β-oxidation by transporting medium- and long-chain fatty acids into the mitochondria [47]. l-carnitine protects against DNA damage caused by the harmful effects of free radicals and maintains a moderate concentration ratio of low acetyl-CoA/CoA [48]. In addition, it also acts as an acetyl storage, providing energy in carbohydrate and lipid metabolic pathways [48]. A recent study demonstrated that l-carnitine has a protective effect via anti-inflammatory and antioxidant mechanisms in the MIA-induced KOA rat model [22]. However, the crucial molecular regulators and the underlying regulatory mechanisms remain unclear. In this study, we reported that l-carnitine supplementation can alleviate synovitis in KOA by regulating lipid accumulation and mitochondrial function. Further mechanistic studies revealed that l-carnitine ameliorates synovitis via the regulation of the AMPK-ACC-CPT1 signaling pathway. In conclusion, our study provides novel therapeutic strategies for the treatment of KOA.

However, some limitations have been identified in this study. First, our investigation of the mechanism of l-carnitine action on the AMPK-ACC-CPT1 signaling pathway in synovial membranes is not sufficiently in-depth and lacks high-quality evidence. Second, clinical trials are necessary to further determine the effectiveness of l-carnitine on the pathogenesis of KOA. Third, it is necessary to set different l-carnitine dose groups and observe more indicators to evaluate the mitochondrial function and lipid metabolism.

## Conclusions

**Fig. 7:**
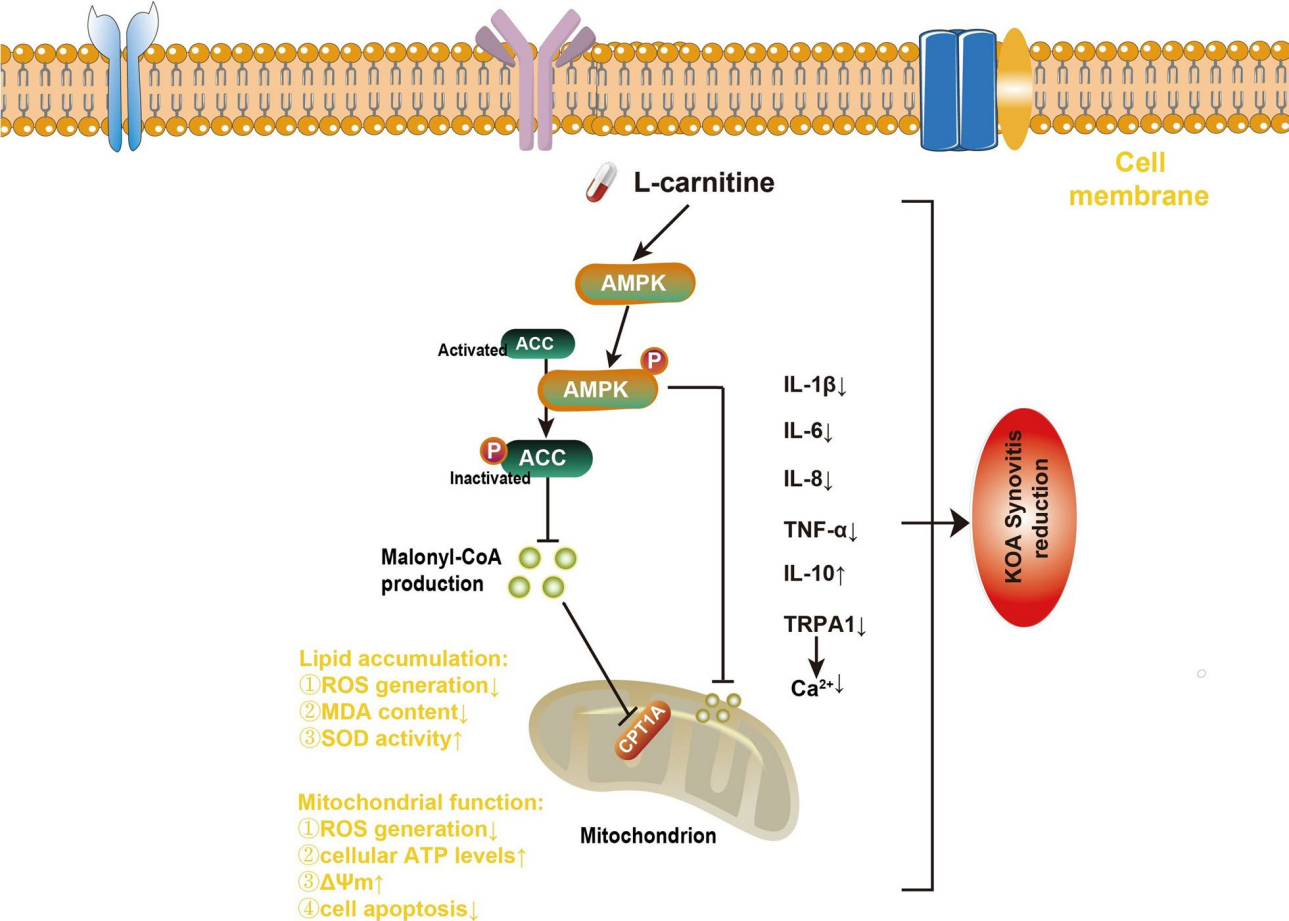
Schematic representation of the role of l-carnitine in the regulation of lipid accumulation and mitochondrial function through the AMPK-ACC-CPT1 signaling pathway in synovial cells (see text for details)

In conclusion, the obtained results indicated the synovioprotective effect of l-carnitine on KOA via the AMPK-ACC-CPT1 signaling pathway, which is linked to mitochondrial function and lipid accumulation (Fig. 7). Hence, we propose that l-carnitine may be considered an effective molecule to protect against synovitis in KOA.

## Data Availability

The datasets used and analyzed during the current study are available from the corresponding author upon reasonable request.

## Abbreviations

KOA: Knee osteoarthritis
LPS: Lipopolysaccharide
FLS: Fibroblast-like synoviocytes
AMPK: AMP-activated protein kinase
CPT1: Carnitine palmitoyltransferase 1
Cyc: Cytochrome c
ACLT: Anterior cruciate ligament transection
NSAIDs: Non-steroidal anti-inflammatory drugs
CoA: Coenzyme A
SOD: Superoxide dismutase
MDA: Malonaldehyde
ATP: Adenosine-triphosphate
ROS: Reactive oxygen species
CCK-8: Cell counting kit-8
DMEM: Dulbecco’s modified Eagle’s medium
IL-1β: Interleukin-1β
siRNA: Small interfering RNA

## References

[1] Sharma L (2021). Osteoarthritis of the Knee. N Engl J Med.

[2] Hunter DJ, March L, Chew M (2020). Osteoarthritis in 2020 and beyond: a lancet commission. Lancet.

[3] Martel-Pelletier J, Barr AJ, Cicuttini FM, Conaghan PG, Cooper C, Goldring MB, Goldring SR, Jones G, Teichtahl AJ, Pelletier JP (2016). Osteoarthritis. Nat Rev Dis Primers.

[4] Turkiewicz A, Petersson IF, Bjork J, Hawker G, Dahlberg LE, Lohmander LS, Englund M (2014). Current and future impact of osteoarthritis on health care: a population-based study with projections to year 2032. Osteoarthr Cartil.

[5] Hunter DJ, Bierma-Zeinstra S (2019). Osteoarthritis. Lancet.

[6] Kan HS, Chan PK, Chiu KY, Yan CH, Yeung SS, Ng YL, Shiu KW, Ho T (2019). Non-surgical treatment of knee osteoarthritis. Hong Kong Med J.

[7] Jang S, Lee K, Ju JH (2021). Recent updates of diagnosis, pathophysiology, and treatment on osteoarthritis of the knee. Int J Mol Sci.

[8] Hochberg MC, Altman RD, April KT, Benkhalti M, Guyatt G, Mcgowan J, Towheed T, Welch V, Wells G, Tugwell P (2012). American College of Rheumatology 2012 recommendations for the use of nonpharmacologic and pharmacologic therapies in osteoarthritis of the hand, hip, and knee. Arthritis Care Res.

[9] Bannuru RR, Osani MC, Vaysbrot EE, Arden NK, Bennell K, Bierma-Zeinstra SMA, Kraus VB, Lohmander LS, Abbott JH, Bhandari M, Blanco FJ, Espinosa R, Haugen IK, Lin J, Mandl LA, Moilanen E, Nakamura N, Snyder-Mackler L, Trojian T, Underwood M, Mcalindon TE (2019). Oarsi guidelines for the non-surgical management of knee, hip, and polyarticular osteoarthritis. Osteoarthr Cartil.

[10] Loef M, Schoones JW, Kloppenburg M, Ioan-Facsinay A (2019). Fatty acids and osteoarthritis: different types, different effects. Jt Bone Spine.

[11] Lee SW, Rho JH, Lee SY, Chung WT, Oh YJ, Kim JH, Yoo SH, Kwon WY, Bae JY, Seo SY, Sun H, Kim HY, Yoo YH (2018). Dietary fat-associated osteoarthritic chondrocytes gain resistance to lipotoxicity through PKCK2/STAMP2/FSP27. Bone Res.

[12] Clockaerts S, Van Osch GJ, Bastiaansen-Jenniskens YM, Verhaar JA, Van Glabbeek F, Van Meurs JB, Kerkhof HJ, Hofman A, Stricker BH, Bierma-Zeinstra SM (2012). Statin use is associated with reduced incidence and progression of knee osteoarthritis in the Rotterdam study. Ann Rheum Dis.

[13] Faas MM, de Vos P (2020). mitochondrial function in immune cells in health and disease. Biochim Biophys Acta Mol Basis Dis.

[14] Mao X, Fu P, Wang L, Xiang C (2020). Mitochondria: potential targets for osteoarthritis. Front Med.

[15] Blanco FJ, Rego I, Ruiz-Romero C (2011). The role of mitochondria in osteoarthritis. Nat Rev Rheumatol.

[16] Li J, Zhang B, Liu WX, Lu K, Pan H, Wang T, Oh CD, Yi D, Huang J, Zhao L, Ning G, Xing C, Xiao G, Liu-Bryan R, Feng S, Chen D (2020). Metformin limits osteoarthritis development and progression through activation of AMPK signalling. Ann Rheum Dis.

[17] Fang K, Wu F, Chen G, Dong H, Li J, Zhao Y, Xu L, Zou X, Lu F (2019). Diosgenin ameliorates palmitic acid-induced lipid accumulation via Ampk/Acc/Cpt-1a and Srebp-1C/Fas signaling pathways in Lo2 cells. Bmc Complement Altern Med.

[18] Schlaepfer IR, Joshi M (2020). Cpt1a-mediated fat oxidation, mechanisms, and therapeutic potential. Endocrinology.

[19] Miguel V, Tituana J, Herrero JI, Herrero L, Serra D, Cuevas P, Barbas C, Puyol DR, Marquez-Exposito L, Ruiz-Ortega M, Castillo C, Sheng X, Susztak K, Ruiz-Canela M, Salas-Salvado J, Gonzalez M, Ortega S, Ramos R, Lamas S (2021). Renal tubule CPT1A overexpression protects from kidney fibrosis by restoring mitochondrial homeostasis. J Clin Investig.

[20] Pekala J, Patkowska-Sokola B, Bodkowski R, Jamroz D, Nowakowski P, Lochynski S, Librowski T (2011). L-carnitine–metabolic functions and meaning in humans life. Curr Drug Metab.

[21] Yi D, Yu H, Lu K, Ruan C, Ding C, Tong L, Zhao X, Chen D (2021). Ampk signaling in energy control, cartilage biology, and osteoarthritis. Front Cell Dev Biol.

[22] Khodir SA, Al-Gholam MA, Salem HR (2020). L-carnitine potentiates the anti-inflammatory and antinociceptive effects of diclofenac sodium in an experimentally-induced knee osteoarthritis rat model. Iran J Basic Med Sci.

[23] Li M, Zhang L, Liu Z, Zhang L, Xing R, Yin S, Li X, Zhang N, Wang P (2021). Sanse powder essential oil nanoemulsion negatively regulates trpa1 by Ampk/Mtor signaling in synovitis: knee osteoarthritis rat model and fibroblast-like synoviocyte isolates. Mediat Inflamm.

[24] Yin S, Wang P, Xing R, Zhao L, Li X, Zhang L, Xiao Y (2018). Transient receptor potential ankyrin 1 (Trpa1) mediates lipopolysaccharide (Lps)-induced inflammatory responses in primary human osteoarthritic fibroblast-like synoviocytes. Inflammation.

[25] Liao T, Ding L, Wu P, Zhang L, Li X, Xu B, Zhang H, Ma Z, Xiao Y, Wang P (2020). Chrysin attenuates the Nlrp3 inflammasome cascade to reduce synovitis and pain in koa rats. Drug Des Devel Ther.

[26] Zhang L, Zhang L, Huang Z, Xing R, Li X, Yin S, Mao J, Zhang N, Mei W, Ding L, Wang P (2019). Increased Hif-1Alpha in knee osteoarthritis aggravate synovial fibrosis via fibroblast-like synoviocyte pyroptosis. Oxid Med Cell Longev.

[27] Liu Z, Liao T, Yang N, Ding L, Li X, Wu P, Wang P (2021). Interventional effects of the topical of "sanse powder" essential oils nanoemulsion on knee osteoarthritis in rats by targeting the ERS/TXNIP/NLRP3 signaling axis. Front Pharmacol.

[28] Fan KJ, Wu J, Wang QS, Xu BX, Zhao FT, Wang TY (2020). Metformin inhibits inflammation and bone destruction in collagen-induced arthritis in rats. Ann Transl Med.

[29] Krenn V, Morawietz L, Haupl T, Neidel J, Petersen I, Konig A (2002). Grading of chronic synovitis—a histopathological grading system for molecular and diagnostic pathology. Pathol Res Pract.

[30] Zhao LR, Xing RL, Wang PM, Zhang NS, Yin SJ, Li XC, Zhang L (2018). NLRP1 and NLRP3 inflammasomes mediate LPS/ATP-induced pyroptosis in knee osteoarthritis. Mol Med Rep.

[31] Geethangili M, Lin CW, Mersmann HJ, Ding ST (2021). Methyl brevifolincarboxylate attenuates free fatty acid-induced lipid metabolism and inflammation in hepatocytes through AMPK/NF-kappaB signaling pathway. Int J Mol Sci.

[32] Schlaepfer IR, Glode LM, Hitz CA, Pac CT, Boyle KE, Maroni P, Deep G, Agarwal R, Lucia SM, Cramer SD, Serkova NJ, Eckel RH (2015). Inhibition of lipid oxidation increases glucose metabolism and enhances 2-deoxy-2-[(18)F]fluoro-D-glucose uptake in prostate cancer mouse xenografts. Mol Imaging Biol.

[33] Zheng L, Zhang Z, Sheng P, Mobasheri A (2021). The role of metabolism in chondrocyte dysfunction and the progression of osteoarthritis. Ageing Res Rev.

[34] Morigny P, Boucher J, Arner P, Langin D (2021). Lipid and glucose metabolism in white adipocytes: pathways, dysfunction and therapeutics. Nat Rev Endocrinol.

[35] Han D, Fang Y, Tan X, Jiang H, Gong X, Wang X, Hong W, Tu J, Wei W (2020). The emerging role of fibroblast-like synoviocytes-mediated synovitis in osteoarthritis: an update. J Cell Mol Med.

[36] Li Q, Lai X, Sun L, Cao J, Ling C, Zhang W, Xiang L, Chen R, Li D, Sun S (2020). Antiobesity and anti-inflammation effects of hakka stir-fried tea of different storage years on high-fat diet-induced obese mice model via activating the AMPK/ACC/CPT1 Pathway. Food Nutr Res.

[37] Kunanusornchai W, Witoonpanich B, Tawonsawatruk T, Pichyangkura R, Chatsudthipong V, Muanprasat C (2016). Chitosan oligosaccharide suppresses synovial inflammation Via AMPK activation: an in vitro and in vivo study. Pharmacol Res.

[38] Misheva M, Kotzamanis K, Davies LC, Tyrrell VJ, Rodrigues P, Benavides GA, Hinz C, Murphy RC, Kennedy P, Taylor PR, Rosas M, Jones SA, Mclaren JE, Deshpande S, Andrews R, Schebb NH, Czubala MA, Gurney M, Aldrovandi M, Meckelmann SW, Ghazal P, Darley-Usmar V, White DA, O'Donnell VB (2022). Oxylipin metabolism is controlled by mitochondrial beta-oxidation during bacterial inflammation. Nat Commun.

[39] Chen P, Zhang Q, Zhang H, Gao Y, Zhou Y, Chen Y, Guan L, Jiao T, Zhao Y, Huang M, Bi H (2021). Carnitine palmitoyltransferase 1c reverses cellular senescence of MRC-5 fibroblasts via regulating lipid accumulation and mitochondrial function. J Cell Physiol.

[40] Li P, Xia Z, Kong W, Wang Q, Zhao Z, Arnold A, Xu Q, Xu J (2021). Exogenous L-carnitine ameliorates burn-induced cellular and mitochondrial injury of hepatocytes by restoring CPT1 activity. Nutr Metab.

[41] Song J, Ren P, Zhang L, Wang XL, Chen L, Shen YH (2010). Metformin reduces lipid accumulation in macrophages by inhibiting foxo1-mediated transcription of fatty acid-binding protein 4. Biochem Biophys Res Commun.

[42] El-Sharaky AS, Wahby MM, Bader EM, Fawzy RA, El-Shahawy IN (2009). Mutual anti-oxidative effect of gossypol acetic acid and gossypol-iron complex on hepatic lipid peroxidation in male rats. Food Chem Toxicol.

[43] Cheleschi S, Gallo I, Barbarino M, Giannotti S, Mondanelli N, Giordano A, Tenti S, Fioravanti A (2019). Microrna mediate visfatin and resistin induction of oxidative stress in human osteoarthritic synovial fibroblasts via Nf-KappaB pathway. Int J Mol Sci.

[44] Tsikas D (2017). Assessment of lipid peroxidation by measuring malondialdehyde (MDA) and relatives in biological samples: analytical and biological challenges. Anal Biochem.

[45] Wachter S, Vogt M, Kreis R, Boesch C, Bigler P, Hoppeler H, Krahenbuhl S (2002). Long-term administration of L-carnitine to humans: effect on skeletal muscle carnitine content and physical performance. Clin Chim Acta.

[46] Rebouche CJ (1992). Carnitine function and requirements during the life cycle. FASEB J.

[47] Dunning KR, Cashman K, Russell DL, Thompson JG, Norman RJ, Robker RL (2010). Beta-oxidation is essential for mouse oocyte developmental competence and early embryo development. Biol Reprod.

[48] Infante JP, Tschanz CL, Shaw N, Michaud AL, Lawrence P, Brenna JT (2002). Straight-chain acyl-CoA oxidase knockout mouse accumulates extremely long chain fatty acids from alpha-linolenic acid: evidence for runaway carousel-type enzyme kinetics in peroxisomal beta-oxidation diseases. Mol Genet Metab.

